# Applying deep learning to right whale photo identification

**DOI:** 10.1111/cobi.13226

**Published:** 2018-11-28

**Authors:** Robert Bogucki, Marek Cygan, Christin Brangwynne Khan, Maciej Klimek, Jan Kanty Milczek, Marcin Mucha

**Affiliations:** ^1^ deepsense.ai Krancowa 5 02–493 Warsaw Poland; ^2^ Institute of Informatics The University of Warsaw Banacha 2 02‐097 Warsaw Poland; ^3^ National Oceanic and Atmospheric Administration Northeast Fisheries Science Center Woods Hole MA 02543 U.S.A.

**Keywords:** algorithm, automated image recognition, computer vision, convolutional neural networks, Kaggle competition, machine learning, photo identification, algoritmo, aprendizaje automático, competencia Kaggle, identificación fotográfica, reconocimiento automatizado de imágenes, redes neurales convolucionales, visión computarizada, 自动图像识别, 计算机视觉, 机器学习, 算法, 卷积神经网络, Kaggle 网站竞赛, 照片识别

## Abstract

Photo identification is an important tool for estimating abundance and monitoring population trends over time. However, manually matching photographs to known individuals is time‐consuming. Motivated by recent developments in image recognition, we hosted a data science challenge on the crowdsourcing platform Kaggle to automate the identification of endangered North Atlantic right whales (Eubalaena glacialis). The winning solution automatically identified individual whales with 87% accuracy with a series of convolutional neural networks to identify the region of interest on an image, rotate, crop, and create standardized photographs of uniform size and orientation and then identify the correct individual whale from these passport‐like photographs. Recent advances in deep learning coupled with this fully automated workflow have yielded impressive results and have the potential to revolutionize traditional methods for the collection of data on the abundance and distribution of wild populations. Presenting these results to a broad audience should further bridge the gap between the data science and conservation science communities.

## Abstract 2

*Article impact statement*: Convolutional neural networks identified region, rotated, and cropped images, standardized photos, and identified correctly individual whales.

## Abstract (es)

Aplicación del Aprendizaje Profundo a la Identificación Fotográfica de la Ballena Franca

**Resumen**

La identificación fotográfica es una herramienta importante para la estimación de la abundancia y el monitoreo de las tendencias poblacionales en el tiempo. Sin embargo, corresponder las fotografías con los individuos conocidos requiere de mucho tiempo. Motivados por los avances recientes en el reconocimiento de imágenes, decidimos acoger un reto de datos científicos en la plataforma de colaboración masiva Kaggle para automatizar la identificación de ballenas francas del Atlántico norte (Eubalaena glacialis), especie que se encuentra en peligro de extinción. La solución ganadora identificó automáticamente a las ballenas individuales con una certeza del 87% y con una serie de redes neurales convolucionales para identificar la región de interés en una imagen, rotar, recortar, y crear fotografías estandarizadas de tamaño y orientación uniforme y después identificar al individuo correcto a partir de estas fotografías tamaño pasaporte. Los avances recientes en el aprendizaje profundo acoplados a este flujo de trabajo completamente automatizado han producido resultados impresionantes y tienen el potencial para revolucionar los métodos tradicionales de recolección de datos de abundancia y distribución de las poblaciones silvestres. La presentación de estos resultados ante un público amplio debería reducir aún más el vacío que existe entre los datos científicos y las comunidades científicas para la conservación.

## 摘要

照片识别是评估种群丰度和监测种群动态的重要工具。然而, 人工地将照片与已知个体进行比对需耗费大量时间。受到最近图像识别领域发展的启发, 我们在众包平台 Kaggle 网站举办了一项数据科学挑战, 来自动识别濒危的北太平洋露脊鲸 (*Eubalaena glacialis)* 。获胜的方案能以87%的准确率自动识别鲸鱼个体, 它利用一系列卷积神经网络来找到图像上关注的区域, 加以旋转、裁剪, 并创建统一尺寸和方向的标准化照片, 然后从这些类似护照的照片中正确识别出鲸鱼个体。目前深度学习领域的进展加上这种完全自动化的工作流程, 已取得了显著成果, 并有可能给野生动物种群丰度和分布的传统数据收集方法带来变革。我们将这些结果呈现给广大受众, 以期进一步缩小数据科学和保护科学群体之间的距离。**【翻译: 胡怡思; 审校: 聂永刚】**

## Introduction

### Photo Identification

Photo identification plays an important role in the field of conservation science. Managing the recovery of endangered species relies on estimating population abundance and monitoring trends over time. Because it is rarely possible to simply count individuals, a common method for estimating abundance is mark recapture (Eberhardt 1969; Otis et al. 1978; Seber 1982). When a repeated sample is taken, a population estimate can be obtained by assuming that the proportion of marked animals caught is proportional to the number of marked individuals in the population. Photo identification is a less invasive approach in which natural markings are used to distinguish between individuals without the stress of capture (Katona & Whitehead 1981; Agler et al. 1990; Würsig & Jefferson 1990). Monitoring wild populations through photo identification allows for the detection of increasing or decreasing abundance trends to inform effective conservation.

The challenge of photo identification to inform conservation is that it is time‐consuming. The era of digital photography has exponentially increased the volume of images submitted to catalogs around the world and has resulted in processing backlogs. Recent advances in machine learning and deep learning in particular have paved the way to automated image processing through the use of neural networks modeled on the human brain. Harnessing this new technology has the potential to revolutionize the speed at which these images can be matched to known individuals.

### North Atlantic Right whales

**Figure 1:**
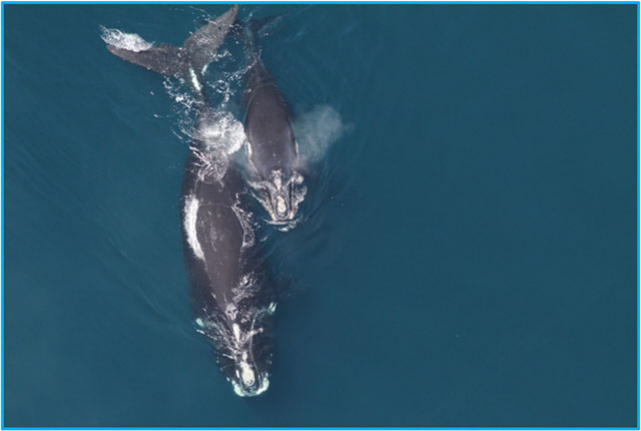
Aerial photograph of a North Atlantic right whale (Eubalaena glacialis) mother and calf showing distinguishing characteristics, including the lack of dorsal fin, stocky black body, and a v‐shaped blow. Image collected under Marine Mammal Protection Act research permit number 17355. Photo credit: National Oceanic and Atmospheric Administration, Northeast Fisheries Science Center, Christin Khan.

North Atlantic right whales (*Eubalaena glacialis*) are large baleen whales well suited to photo identification because they can be individually identified by the callosity pattern on the top of their heads (Fig. 1) (Kraus et al. 1986; Hamilton & Martin 1999). Callosities are patches of rough skin colonized by tiny crustaceans (whale lice) that result in a distinctive white pattern against the otherwise black body (Payne 1976). Researchers take photographs from vessels, drones, and aircraft and match individuals to the North Atlantic Right Whale Catalog (New England Aquarium 2017). The long‐term nature of this data set allows for a nuanced understanding of demographics, social structure, reproductive rates, individual movement patterns, genetics, health, and anthropogenic mortality. Despite international protection since 1935, right whales have been slow to recover due to entanglement in fishing gear and ship collisions (van der Hoop et al. 2013; Henry et al. 2016). Conservation efforts have included vessel speed restrictions (Clapham & Pace 2001; Silber et al. 2014), modification of international shipping lanes (Vanderlaan et al. 2008; Wiley et al. 2013), aircraft and vessel monitoring surveys (Cole et al. 2007; Khan et al. 2016), right whale alerts to mariners (Cole et al. 2007), the Mandatory Ship Reporting system (Silber et al. 2015), stranding response, and outreach efforts. Photo identification data was used in a state‐space mark‐recapture model to estimate there were only 451 North Atlantic right whales remaining in 2016 and that the species has been declining since 2010 (Waring et al. 2016; Pace et al. 2017).

### Competition and Collaboration

Data science competitions connect data problems to data solutions by crowdsourcing. The benefit is that a wide variety of strategies can be applied to produce the best models for predicting and describing data sets to see which methods yield the most promising results. Casting a wide net is particularly valuable when solving complex problems that demand creative approaches. Platforms such as Kaggle and TopCoder attract a large talent pool by providing large sets of cleaned and annotated data ready to use for testing the latest machine‐learning approaches. The leaderboards have become central to career development and job placement in the data science industry which brings the best and brightest competitors to the table. Motivated by the recent advances in image recognition combined with the ability to crowd source a wide variety of approaches to tackle this complex problem, the National Oceanic and Atmospheric Administration hosted a data science challenge on Kaggle to automate the identification of North Atlantic right whales. This publication is the result of a collaboration that formed after the competition between the data scientists who won the competition and the biologist who organized it. We present the winning solution, which automatically identifies individual right whales with 87% accuracy and promises to speed up the process of photo identification.

### Machine Learning

There have been previous applications of computer vision to recognition of individuals (e.g., Arzoumanian et al. 2005; Crall et al. 2013; Beijbom et al. 2015), including whales (e.g., Hiby & Lovell 2001; Kniest 2010; Flukebook 2018). Most approaches start by extracting certain features of the image that are then used to train the machine‐learning model. The features are typically obtained by applying standard filters to the input image (e.g., Gabor filters, DoG filters, etc.) with the choice of filters based on experimentation and/or experience from manual individual recognition. Another common characteristic of existing approaches is that a sequence of tasks is typically performed manually, such as selecting key points, rotating, aligning, and cropping. In most cases such tasks can be performed in a matter of minutes or even seconds; however, the necessity of this delay may interfere with other urgent actions when used on the scene.

The machine‐learning schema consists of designing the mathematical structure of a model and an objective function to be optimized, training the model or models, evaluating model quality, and selecting the best performing model (Russell & Norvig 1995). There are several types of machine‐learning algorithms including supervised learning, reinforcement learning, and unsupervised learning. We focused on supervised learning with a ground‐truthed training data set (e.g., images were labeled with the correct whale identification).

Neural networks are an established family of machine‐learning models inspired by the human brain. Recently neural networks, and in particular convolutional neural networks (CNN), have had several breakthrough developments and spectacular applications (e.g., beating professional human players in the game of GO) (Silver et al. 2016). They have become the model of choice for image processing applications since the groundbreaking work of Krizhevsky et al. (2012) and have been very effective in image classification (He et al. 2016), image segmentation (Long et al. 2015), object detection (Ren et al. 2015; Redmon et al. 2016), face recognition (Taigman et al. 2014), and microscopy (Xing et al. 2017). The big improvements driving these new developments come from mathematical understanding of the learning process of neural‐network‐based models and rapid development in computing capabilities of graphical processing units (e.g., Oh & Jung 2004; Raina et al. 2009; Cireşan et al. 2010).

**Figure 2:**
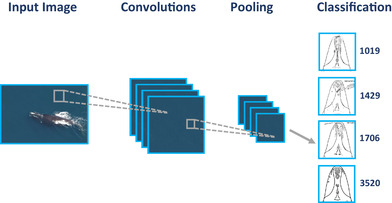
Convolutional neural network such as the one used to identify individual North Atlantic right whales. Each layer (squares) within a network receives an input image, performs a transformation on the image, and outputs the results to the subsequent layer. Images collected under Marine Mammal Protection Act research permit number 17355. Photo credit: National Oceanic and Atmospheric Administration, Northeast Fisheries Science Center, Christin Khan. Drawings from Anderson Cabot Center for Ocean Life at the New England Aquarium, used with permission.

As the number of layers (Fig. 2) in the state‐of‐the‐art convolutional neural networks increased, the term “deep learning” was coined as a phrase denoting training a neural network with many layers (Aizenberg et al. 2000; Goodfellow et al. 2016). Each layer receives an input image, performs a transformation and outputs the results to the subsequent layer. The input to the first layer is the original image itself, which can be viewed as a 2‐dimensional array of pixels, where for each pixel 3 values are stored (red, green, blue). For example, consider a layer that takes an input image and scales the image down by a factor of 2 in each dimension. Such a layer would split the input image into small disjoint regions of 2 × 2 pixels size, and compute the average intensity for each of the regions. This is an example of a pooling layer. The pooling layers are defined by a set of parameters, such as the size of the small region, whether they overlap slightly or are disjoint, whether an average or maximum intensity should be computed.

The most important layers in convolutional neural networks are the convolutional layers themselves. A convolutional layer computes a new value for each pixel. The new value of a pixel depends not only on the old value of that pixel but also on the old values of nearby pixels. For example, assume the new value of a pixel is computed by subtracting neighboring left pixel value from the old value of the pixel in question. Applying such an operation to all the pixels of an image results in an edge detector; intuitively, pixels with similar‐valued neighbors will get small values, whereas pixels lying on a sharp boundary will be assigned a large value. A wide variety of such transformations can be defined depending on the exact formula for computing the new pixel's value. An important characteristic of convolutions is that they are translation invariant, meaning that 2 parts of an image that are the same will also remain the same after transformation.

A convolutional neural network consists of a sequence of convolutional, pooling, and other layers that together form a complex transformation. The exact behavior of the transformation depends on the parameters of particular layers. A single layer of a CNN can be viewed as a kernel convolution filter, as used in computer vision, and the whole network is a pipeline of such filters. The key feature of CNNs is that the parameters of the filters are automatically optimized to the specific task being solved. Another important detail in the process of an image going through the network is that the channels lose their connection to colors (red, green, blue) and represent some abstract notion of features.

## Methods

### Whale Photographs and Algorithm development

**Figure 3:**
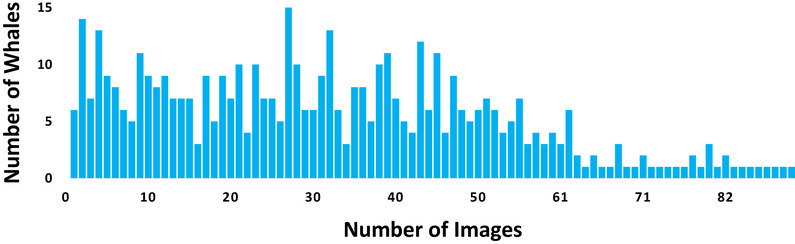
Number of images per whale in the Kaggle competition data set of North Atlantic right whales (Eubalaena glacialis) .

**Figure 4:**
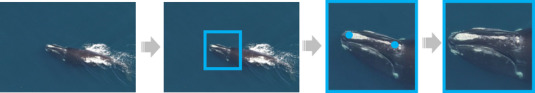
Our workflow pipeline from the original photograph to a standardized right whale passport photograph (i.e., photos have a uniform size and orientation) (first photo, original high‐resolution aerial photograph; second photo, region of interest localized by identifying a bounding box around the head of the whale; third photo, image cropped based on region and new, more precise key points located in the cropped image and used to rotate the image; fourth photo, standardized right whale photo with uniform size and orientation). Images collected under Marine Mammal Protection Act research permit number 17355. Photo credit: National Oceanic and Atmospheric Administration, Northeast Fisheries Science Center, Christin Khan.

Photographs were obtained from the National Oceanic and Atmospheric Administration, which conducts aerial surveys to monitor the abundance and distribution of North Atlantic right whales. The photographs were matched to the North Atlantic Right Whale Consortium photo identification catalog (New England Aquarium 2017) to confirm the individual identification (Hamilton & Martin 1999). The training data set provided for the Kaggle competition consisted of 4544 images that contained only 1 single right whale and was labeled with a correct whale identification. Additionally, there was a set of 4111 images, for which a team could submit their predictions during the contest to get an aggregated score as a feedback to inform algorithm development. Submissions were evaluated on a test set of 2493 images used to determine the winners at the end of the competition. This data set is large by the standards of the wildlife research community but relatively small by the standards of deep learning algorithms. The number of images per whale varied considerably; 6 individuals had only 1 photograph, whereas there were 2 whales that each had 82 images (Fig. 3). Theoretically a single neural network could perform all the actions simultaneously, but design and training of such a network is more difficult, so we split the recognition process into steps: locating the whale, cropping, rotating, and classifying (Fig. 4). Overall, our pipeline did not differ significantly from those of the known systems, with a key exception—all the steps were performed automatically. Given the intended broad audience, we have omitted some of the more detailed methods which can be found in Bogucki et al. (2016).

### Region of Interest

Identifying the region of interest in a photo was an important first step because the typical width and height of the images is of the order of thousands of pixels, but the whale occupies only a tiny fraction of the image. Downscaling the images first would result in significant loss of detail and consequently poor quality cropping and rotating decisions. Therefore, we introduced a preliminary step, in which a CNN roughly selected the region of interest (whale's head) in a scaled down image (down to size 256 × 256) and output a bounding box which was then used to crop the high‐resolution image. To train the network, we manually selected bounding boxes for the training data set. However, in the final algorithm, this was done automatically.

### Rotation and Cropping

Rotating and cropping the images into a standardized format also improved the performance of the algorithms. The CNNs have translation invariance: if a network has learned to identify a certain pattern at 1 location, it can identify it at all locations. However, CNNs do not have scale and rotation invariance. This was generally resolved by normalizing the images so that the objects of interest always had roughly the same size and orientation or augmenting the data set with randomly rotated and scaled copies of images (because the augmented data were rotation and scale invariant, the network learned this invariance as well). Although rotation invariance and scale invariance were natural and desirable properties for a whale identification system, performing these actions also facilitated training the main identification CNN immensely. Normalizing the images simplified the concept to be learned, and augmentation effectively increased the size of the data set. Based on preliminary experiments, we normalized the images and used only moderate data augmentation (Bogucki et al. 2016). We developed a network that automatically scaled, rotated and cropped the input image, producing what we call a passport photo of a whale (Fig. 4). This was achieved by identifying 2 key points on the top of the whale's head at either end of the callosity—the tip of the bonnet and just below the blowholes (Fig. 4). We trained a CNN to locate these key points with annotations provided by A. Thomas at https://github.com/anlthms/whale-2015. Once the key‐point positions were identified, the image was scaled, rotated, and cropped so that the whale's head occupied a predefined position (Fig. 4).

### Data Augmentation

Despite normalizing data to achieve rotation invariance and scale invariance, we used data augmentation as well. The normalization process was not perfect, and there were still slight variations in the alignment and scale of the whale. This was rectified by adding very slightly rotated and rescaled versions of the images to the data set. We also trained the model to ignore other irrelevant details by applying certain random perturbations of the color space (Krizhevsky et al. 2012), which compensated for variations in the color and texture of the images due to variations in weather, camera equipment, aircraft orientation, and sun angle.

### Label Augmentation, Network Architecture, and Retraining

Another way in which subtasks were introduced into the system architecture was with label augmentation, which forced the CNN to perform more than just whale identification by answering simple questions (e.g., Did the callosities form a connected pattern? Was the whale oriented dorsal side up? Or, was the image of sufficient quality?). Introducing additional labels did not compromise the performance of the CNN because, although the sheer amount of information increased, it became significantly more structured. By introducing additional labels, we incentivized the network to learn useful concepts which made the learning process faster. We used callosity connectivity augmentation, which was the only label to improve performance. The training images were manually inspected and scored for whether or not the callosity was connected.

We used 3 different CNNs to build our model—1 identified the region of interest, 1 identified key points on the head of the whale, and 1 identified the whale to the correct individual. The CNN used to identify the region of interest used 5 convolutional layers interspersed with 5 pooling layers, followed by a fully connected layer. The CNN used to identify the key points on the head of the whale (to align the image) used 9 convolutional layers, most of them followed by pooling layers, and a fully connected layer. Finally, the most complex CNN was used to perform actual whale identification, which had 11 convolutional layers, 6 pooling layers and a fully connected layer. For each of those 3 tasks, we used several similar networks and then averaged the predictions to improve performance.

Initially, we set aside 10% of the training data to use for internal validation. However, to fully utilize all the data, at the very end we added this validation set back into the training set and performed additional training on all models. Without this added step, the model may not have been able to identify underrepresented whales, for which significant portion of the photos ended up in the validation set. However, training on the complete training data set risked overfitting, so it had to be done carefully (small learning rate, etc.).

### Evaluation

Performance measures such as accuracy or top‐5 accuracy (1 of top 5 predictions is correct) are commonly used to evaluate the success of image recognition (Russakovsky et al. 2015). Although these measures are intuitive, they cannot be used directly as objective functions when training neural networks. This is because the training process proceeds in many small steps, so small that most of them do not affect accuracy at all. Therefore, more fine‐grained performance measures are used to train neural networks that are sensitive to very small changes in the model parameters and guide them in the right direction. These measures are not defined in terms of predicted labels that the model gives for each input image; instead, they are defined in terms of confidences that it assigns to each possible answer from the softmax layer. The most common choice for classification problems is the cross‐entropy loss, also called logloss, which was a natural choice for this work and was used to evaluate winners of the Kaggle competition. Each image was labeled with 1 true class and a set of predicted probabilities was submitted for each whale. The following formula was used:

(1) $$\mathrm{logloss}=-\frac{1}{N}\sum_{i=1}^{N}\;\sum_{j=1}^{M}y_{ij}\log\left(p_{\mathrm{ij}}\right)\;,$$

where *N* was the number of images in the test set, *M* was the number of whale labels, log was the natural logarithm, *y_ij_* was 1 if observation *i* belongs to whale *j* and 0 otherwise, and *p_ij_* was the predicted probability that observation *i* belongs to whale *j*. To avoid the extremes of the log function, predicted probabilities were replaced with maximum probabilities (min [*p*, 1−10^−15^], 10^−15^). Submissions were submitted as a csv file with the image file name, all candidate whale IDs, and a probability for each whale ID on a test set of 2493 images for which the whale identity was not provided to the contest participants.

## Results

The Kaggle challenge attracted considerable attention in the data science community with 364 teams participating. Our model was the winning solution and matched the photograph to the correct individual right whale in 87.44% of cases. Our model yielded 94.87% top‐5 accuracy, which was the number of times the model output the correct whale identification when allowed to output 5 possible whale identifications. The confidence level of a prediction can be indicated with an auxiliary number (between 0 and 1), and cross‐entropy loss is commonly used to measure these confidence levels. On the test set, our solution obtained a cross‐entropy loss of 0.596.

Our machine‐learning model for recognizing individual right whales consisted of a fully automated pipeline utilizing a series of CNNs that identified the region of interest with specific key points on the head, and then rotated and cropped the image to create standardized passport photographs. These passport photographs were then used to identify the correct individual right whale. Our model improved on the limitations associated with previous applications of computer vision for automated recognition with a series of CNNs with no manual input. These networks were in essence sequences of image filters. However, the individual filters were not supplied by the designer, but were instead automatically optimized to best suit a particular application. The open‐source algorithms can be downloaded from GitHub: https://github.com/robibok/whales.

The training of our final architecture including all substeps took 7 days on a single NVIDIA K80 graphics processing unit. Once the training process finished, the models were ready to be queried for predictions. The time required for processing a single photo can vary from tens of seconds to a tenth of a second depending on machine's computational power, but can be done even on a modern laptop.

## Discussion

The use of a data science competition allowed us to benefit from many different approaches being applied to solve this challenging image recognition problem. The winning solution in the Kaggle contest devised by the team from Deepsense.ai had significant advances over existing approaches. First, all steps in the classification process were fully automated, so there was no need for user input. Second, we built on the significant advances in the field of computer vision since the groundbreaking work of Krizhevsky et al. (2012). The fully automated pipeline used a series of convolutional neural networks to identify the region of interest with specific key points on the head and then to rotate and crop the image to create standardized photographs. These photographs were then used to identify the correct individual right whale.

### Challenges

Images representing different classes (i.e., individual whales) were very similar to each other, in contrast to the situation where the algorithm discriminates between objects such as dogs, cats, and airplanes. This posed some difficulties for the neural networks—the unique characteristics that set a particular whale apart from others occupied only a small portion of an image and were not very apparent. On our way to the final solution, we tested many different approaches mostly related to the creation of a complete end‐to‐end system without the need for intermediate steps such as the detection of key points or a region of interest. Future developments in deep learning have the potential to produce such an end‐to‐end system without the need for multiple steps.

**Figure 5:**
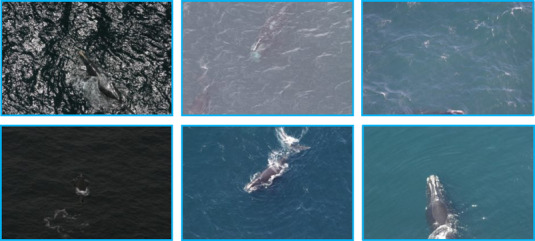
Sample photographs from the data set of North Atlantic right whales showing the variation in quality and problems (from right to left and top to bottom): sun glare; overexposed; white caps due to wind; underexposed, white water from whale movement, and partially visible whale. Images collected under Marine Mammal Protection Act research permit number 17355. Photo credit: National Oceanic and Atmospheric Administration, Northeast Fisheries Science Center, Christin Khan.

Although there was a wide variability in the number of images per individual whale (Fig. 3), this did not seem to affect the training process—the system learned how to recognize even the whales with low number of sample images. Only the last step of our pipeline (whale recognition from a passport photograph) was affected by the nonuniform frequencies. Finding the region of interest and key points was performed by a model trained on the amassed collection of all the whales. Although having more images per individual could improve accuracy, data augmentation would compensate for a smaller data set. We inspected the 313 misclassified images from the test set and did not find anything special. Accuracy could likely be improved by excluding challenging images such as those where the whale was only partially visible or with particularly challenging lighting conditions (Fig. 5). However, this improvement in accuracy would come at the cost of designing a system with more stringent photograph quality requirements, which may not be as desirable for the user. Furthermore, the vast majority of the images look superficially similar to the ones correctly classified. For this reason, we believe there is still room for improvement in this challenging task.

### Relevance to Other Species

It is standard for image classification problems to assume a fixed set of possible labels and in this case, we used a closed population of North Atlantic right whales. Additional labels could be introduced by retraining on additional images either the whole pipeline or some parts of it. Given recent successful applications of deep learning methods to face recognition, we expect our system can handle significantly larger populations and still deliver accurate predictions (such as the closely related Southern right whale which has thousands of individuals). To expand this work beyond right whales, the system would require additional customization to identify the key points and generate region of interest labeling to reflect the individually distinctive features of that species. So, although our model architecture can serve as a solid starting point for related problems, there is not yet a single solution that can be directly applied to other species without any modification.

### Quality Assurance

As scientists increasingly rely on automated image recognition, verification will be needed to ensure the continued quality of photo‐identification catalogs. Humans have a tendency to rely too heavily on the first solution presented to them, a cognitive bias known as anchoring, which could lead to false identifications. While this bias can influence the process of manual photo matching, it has the potential to be even stronger when presented with a high confidence score from an algorithm. Given the importance of accurate photo identification data to inform estimates of survival, abundance, and reproductive rates, we urge data managers to continue the process of independent verification to ensure that new methods are performing as expected.

### Next Steps

We plan to package these algorithms into the user‐friendly platform Flukebook in collaboration with Wild Me, a nonprofit organization that blends structured wildlife research with artificial intelligence, citizen science, and computer vision to speed population analysis and develop new insights to help fight extinction (Flukebook 2018). Marine biologists with no background in machine learning will be able to automatically identify individual right whales by uploading images to a website and then receiving suggested matches along with a percent confidence score. The algorithms will be run on a secure cloud services platform so the only user requirement is a reliable internet connection and no need for high end graphics processing units. This collaboration will bring the greatest strengths of each organization together to form the best solution for automated image recognition of this important species.

The collaborative approach we took applies state‐of‐the‐art image recognition to a conservation science problem. The recent advances in the field of deep learning coupled with this fully automated workflow have yielded impressive results and have the potential to revolutionize traditional data collection methods for the abundance and distribution of wild populations. Streamlining the process of photo identification could increase the efficiency of research on wild populations, enable cross‐matching between existing catalogs, and allow for the processing of larger volumes of data, particularly when coupled with a workflow that can automate the processing of images and video taken from aircraft, drones, submersibles, or camera traps. Presenting these results to a broad audience should further bridge the gap between the data science and conservation science communities and encourage others to adopt these approaches for application in other species.

## References

[cobi13226-bib-0001] Agler BA , Beard JA , Bowman RS , Corbett HD , Frohock SE , Hawvermale MP , Katona SK , Sadove SS , Seipt IE . 1990 Fin whale (*Balaenoptera physalus*) photographic identification: methodology and preliminary results from the western North Atlantic. Report of the Meeting of the International Whaling Commission 12:349–356.

[cobi13226-bib-0002] Aizenberg I , Aizenberg N , Vandewalle J . 2000 Multi‐valued and universal binary neurons: theory, learning and applications. Springer Science & Business Media, Germany.

[cobi13226-bib-0003] Arzoumanian Z , Holmberg J , Norman B . 2005 An astronomical pattern‐matching algorithm for computer‐aided identification of whale sharks *Rhincodon typus* . Journal of Applied Ecology 42:999–1011.

[cobi13226-bib-0004] Beijbom O , et al. 2015 Towards automated annotation of benthic survey images: variability of human experts and operational modes of automation. PLOS ONE 10 (e0130312) 10.1371/journal.pone.0130312. PMC4496057 26154157

[cobi13226-bib-0005] Bogucki R , Cygan M , Klimek M , Milczek JK , Mucha M . 2016 Which whale is it, anyway? Face recognition for right whales using deep learning. deepsense.ai, Palo Alto, California. Available from https://deepsense.ai/deep-learning-right-whale-recognition-kaggle/.

[cobi13226-bib-0006] Cireşan DC , Meier U , Gambardella LM , Schmidhuber J . 2010 Deep, big, simple neural nets for handwritten digit recognition. Neural Computation 22:3207–3220. 20858131 10.1162/NECO_a_00052

[cobi13226-bib-0007] Clapham PJ , Pace III RM . 2001 Defining triggers for temporary area closures to protect right whales from entanglements: issues and options. Northeast Fisheries Science Center Reference Document 01–06. U.S. Department of Commerce, Washington, D.C.

[cobi13226-bib-0008] Cole TVN , Gerrior P , Merrick RL . 2007 Methodologies and preliminary results of the NOAA National Marine Fisheries Service aerial survey program for right whales (*Eubalaena glacialis*) in the northeast U.S., 1998–2006. Northeast Fisheries Science Center Reference Document 07‐02; U.S. Department of Commerce, Washington, D.C.

[cobi13226-bib-0009] Crall JP , Stewart CV , Berger‐Wolf TY , Rubenstein DI , Sundaresan SR . 2013 HotSpotter ‐ patterned species instance recognition. Proceedings of the IEEE workshop on applications of computer vision. Institute of Electrical and Electronics Engineers, Piscataway, New Jersey.

[cobi13226-bib-0010] Eberhardt LL . 1969 Population estimates from recapture frequencies. 33:28–39.

[cobi13226-bib-0011] Flukebook . 2018 Flukebook helps you study identify, and protect cetacean populations. Flukebook, Portland, Oregon. Available from http://www.flukebook.org/ (accessed November 2018).

[cobi13226-bib-0012] Goodfellow I , Bengio Y , Courville A . 2016 Deep learning. MIT Press, Cambridge, Massachusetts.

[cobi13226-bib-0013] Hamilton PK , Martin SM . 1999 A catalog of identified right whales from the Western North Atlantic: 1935 to 1997. New England Aquarium, Boston.

[cobi13226-bib-0014] He K , Zhang X , Ren S , Sun J . 2016 Deep residual learning for image recognition. The IEEE conference on computer vision and pattern recognition (CVPR) 2016:770–778.

[cobi13226-bib-0015] Henry AG , Cole TVN , Hall L , Ledwell W , Morin D , Reid A . 2016 Serious injury and mortality determinations for baleen whale stocks along the Gulf of Mexico, United States East Coast and Atlantic Canadian Provinces, 2010–2014. Northeast Fisheries Science Center reference document 16‐10. U.S. Department of Commerce, Washington, D.C.

[cobi13226-bib-0016] Hiby L , Lovell P . 2001 A note on an automated system for matching the callosity patterns on aerial photographs of southern right whales. Journal of Cetacean Research and Management 2:291–295.

[cobi13226-bib-0017] Katona S , Whitehead H . 1981 Identifying humpback whales using their natural markings. Polar Record 20:439–444.

[cobi13226-bib-0018] Khan C , Duley P , Henry A , Gatzke J , Crowe L , Cole T . 2016 North Atlantic Right Whale Sighting Survey (NARWSS) and Right Whale Sighting Advisory System (RWSAS) 2014 results summary. Northeast Fisheries Science Center Reference Document 16‐01. U.S. Department of Commerce, Washington, D.C.

[cobi13226-bib-0019] Kniest E. 2010 Fluke Matcher: a computer‐aided matching system for humpback whale (*Megaptera novaeangliae*) flukes. Marine Mammal Science 26:744–756.

[cobi13226-bib-0020] Kraus SD , Moore KE , Price CA , Crone MJ , Watkins WA , Winn HE , Prescott JH . 1986 The use of photographs to identify individual North Atlantic right whales (*Eubalaena glacialis*). Report of the International Whaling Commission 10:145–151.

[cobi13226-bib-0021] Krizhevsky A , Sutskever I , Hinton G . 2012 Imagenet classification with deep convolutional neural networks. Advances in Neural Information Processing Systems (NIPS) 1:1097–1105.

[cobi13226-bib-0022] Long J , Shelhamer E , Darrell T . 2015 Fully convolutional networks for semantic segmentation. The IEEE Conference on Computer Vision and Pattern Recognition, Boston, Massachusetts, pp. 3431–3440. 10.1109/TPAMI.2016.2572683 27244717

[cobi13226-bib-0023] New England Aquarium . 2017 The north Atlantic right whale catalog. New England Aquarium, Boston. Available from http://rwcatalog.neaq.org (accessed March 2017).

[cobi13226-bib-0024] Oh K , Jung K . 2004 GPU implementation of neural networks. Pattern Recognition 37:1311–1314.

[cobi13226-bib-0025] Otis DL , Burnham KP , White GC , Anderson DR . 1978 Statistical inference from capture data on closed animal populations. Wildlife Monographs 62:3–135.

[cobi13226-bib-0026] Pace III RM , Corkeron PJ , Kraus SD . 2017 State–space mark–recapture estimates reveal a recent decline in abundance of North Atlantic right whales. Ecology and Evolution 7:8730–8741. 29152173 10.1002/ece3.3406 PMC5677501

[cobi13226-bib-0028] Payne R. 1976 At home with right whales. National Geographic Magazine 149:322–339.

[cobi13226-bib-0029] Raina R , Madhavan A , Ng AY . 2009 Large‐scale deep unsupervised learning using graphics processors. Proceedings of the 26th International Conference on machine learning, Montreal, Canada.

[cobi13226-bib-0031] Redmon J , Divvala S , Girshick R , Farhadi A . 2016 You only look once: unified, real‐time object detection. The IEEE Conference on Computer Vision and Pattern Recognition, pp. 779–788.

[cobi13226-bib-0030] Ren S , He K , Girshick R , Jian S . 2015 Faster R‐CNN: Towards real‐time object detection with region proposal networks. Advances in Neural Information Processing Systems, pp. 91–99.

[cobi13226-bib-0032] Russakovsky O , et al. 2015 ImageNet large scale visual recognition challenge. International Journal of Computer Vision 115:211–252.

[cobi13226-bib-0033] Russell SJ , Norvig P . 1995 Artificial intelligence: a modern approach. Prentice Hall, Upper Saddle River, New Jersey.

[cobi13226-bib-0034] Seber GA. 1982 The estimation of animal abundance and related parameters. Charles Griffin, London.

[cobi13226-bib-0035] Silber GK , Adams JD , Asaro MJ , Cole TVN , Moore KS , Ward‐Geiger LI , Zoodsma BJ . 2015 The right whale mandatory ship reporting system: a retrospective. PeerJ 3:e866. 25861555 10.7717/peerj.866 PMC4389273

[cobi13226-bib-0036] Silber GK , Adams JD , Fonnesbeck CJ . 2014 Compliance with vessel speed restrictions to protect North Atlantic right whales. PeerJ 2:e399. 24949229 10.7717/peerj.399 PMC4060020

[cobi13226-bib-0037] Silver D , et al. 2016 Mastering the game of go with deep neural networks and tree search. Nature 529:484–489. 26819042 10.1038/nature16961

[cobi13226-bib-0038] Taigman Y , Yang M , Ranzato MA , Wolf L . 2014 DeepFace: closing the gap to human‐level performance in face verification. The IEEE Conference on Computer Vision and Pattern Recognition 2014:1701–1708.

[cobi13226-bib-0039] Van der Hoop JM , Moore MJ , Barco SG , Cole TV , Daoust P , Henry AG , McAlpine DF , McLellan WA , Wimmer T , Solow AR . 2013 Assessment of management to mitigate anthropogenic effects on large whales. Conservation Biology 27:121–133. 23025354 10.1111/j.1523-1739.2012.01934.x PMC3562480

[cobi13226-bib-0040] Vanderlaan ASM , Taggart CT , Serdynska AR , Kenney RD , Brown MW . 2008 Reducing the risk of lethal encounters: vessels and right whales in the Bay of Fundy and on the Scotian Shelf. Endangered Species Research 4:283–297.

[cobi13226-bib-0041] Waring GT , Josephson E , Maze‐Foley K , Rosel PE , editors. 2016 US Atlantic and Gulf of Mexico marine mammal stock assessments‐2015. National Oceanic and Atmospheric Administration (NOAA) technical memorandum NMFS NE 238. NOAA.

[cobi13226-bib-0042] Wiley D , Hatch L , Thompson M , Schwehr K , MacDonald C . 2013 Marine sanctuaries and marine planning: protecting endangered marine life. Proceedings of the Marine Safety & Security Council, the Coast Guard Journal of Safety at Sea. Fall 2013:10–15.

[cobi13226-bib-0043] Würsig B , Jefferson RA . 1990 Methods of photo‐identification for small cetaceans. Report of the International Whaling Commission 12:42–43.

[cobi13226-bib-0044] Xing F , Xie Y , Su H , Liu F , Yang L . 2017 Deep learning in microscopy image analysis: a survey. IEEE Transactions on Neural Networks and Learning Systems 99:1–19. 10.1109/TNNLS.2017.2766168 29989994

